# Data on modeling of nexus between entrepreneurs׳ commitment and business performance in a developing country

**DOI:** 10.1016/j.dib.2018.05.102

**Published:** 2018-05-28

**Authors:** Ayoade Omisade, Ogunnaike Olaleke, Adegbuyi Omotayo, Kehinde Oladele, Iyiola Oluwole, Lawal Fatai, Onakoya Femi

**Affiliations:** Department of Business Management, Covenant University, Ota, Ogun State, Nigeria

## Abstract

This article presents data that examined the modeling of nexus between entrepreneurs׳ commitment and business performance in a developing country. 315 copies of questionnaire were retrieved from 400 copies that were administered to Nigerian Association of Small Scale Industrialists (NASSI) Southwest chapters which comprised of six states. In addition to descriptive analysis of part of the data, correlation and regression analysis were used to present the data through structural equation model (SEM). The SEM path analysis shows the estimates of the interconnectedness of the major constructs in the data.

**Specifications Table**

**Table:** 

Subject area	*Entrepreneurship and Business Management*
More specific subject area	*Entrepreneurs’ Commitment and Business Performance*
Type of data	*Figure, Chart, Table, and SPSS*
How data was acquired	*Questionnaire survey*
Data format	*Raw, analyzed, descriptive and statistical data.*
Experimental factors	*The population and sample where this data was collected consist of registered members of NASSI. Questionnaire was formulated around the three layers of commitment (Affective, Continuous and Normative) by Meyer and Allen (1997) and business performance was measured by customer satisfaction, market share and profit.*
Experimental features	*Entrepreneurs’ commitment is paramount for the survival of business at different cycle of business activities.*
Data source location	*Entrepreneurs in Southwest Nigeria*
Data accessibility	*Data is included in this article.*

**Value of the data**

• The data present the relationship and its impact of entrepreneurs’ commitment on business performance.
• The demographic nature of the entrepreneurs was highlighted by the data.
• The data shows the level of impact each of the commitment variables had on business performance.
• The data will aid further discourse on the interconnectedness of entrepreneurs’ commitment towards business performance.

## 1 Data

**Fig. 1:**
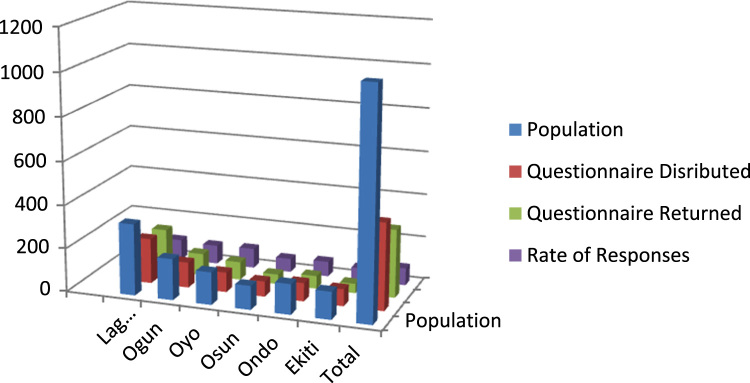
Questionnaire distribution and response rate.

**Fig. 2:**
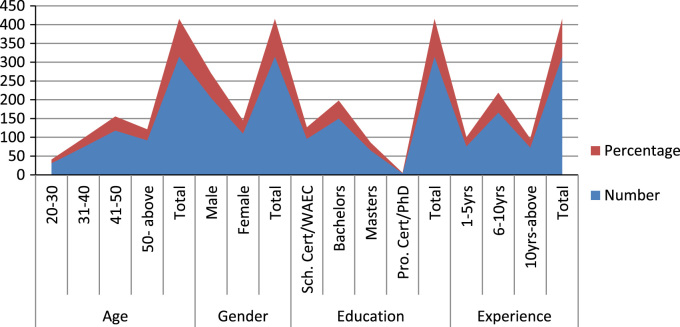
Graphical representations of demographic characteristics of respondents.

**Fig. 3:**
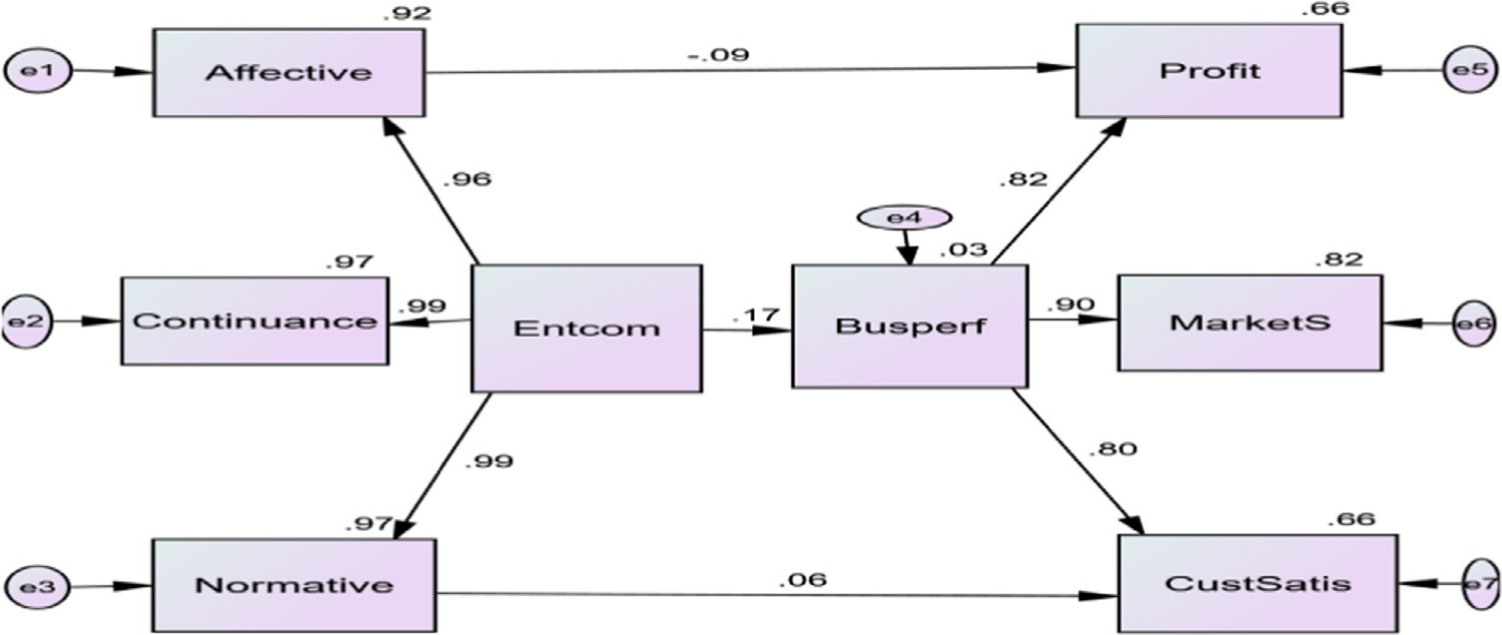
Amos Output. Notes: Affective = Affective Commitment, Continuance = Continuance Commitment, Normative = Normative Commitment, Entcom = Entrepreneurs’ Commitment, Busperf = Business Performance, CusSatis = Customers’ Satisfaction, MarketS = Market Share, Profit = Profit.

The data contained 315 copies of questionnaire retrieved from 400 copies administered to entrepreneurs in six states of Southwest Nigeria. Table 1 and Fig. 1 show the distribution in each of the state and the response rate. Confidentiality of the respondents was guaranteed as names and telephone numbers not requested on the survey. The demographic representation is given in Table 2 and Fig. 2 while Fig. 3 gives the path diagram of the structural equation model. The standardize regression weight and the correlation coefficient is shown in Table 3, Table 4 respectively.

**Table 1:** Questionnaire distribution and response rate.

Name of Branches	Population	Questionnaire distributed	Questionnaire returned	Rate of response %
Lagos	330	208	198	95
Ogun	190	117	102	87
Oyo	150	91	83	91
Osun	110	71	45	63
Ondo	140	85	59	69
Ekiti	127	78	44	56
Total	1047	400	315	79

**Table 2:** Demographic characteristics of respondents.

**Demographic**	**Description**	**Number**	**Percentage**
**Age**	20–30	31	9.8
	31–40	74	23.5
	41–50	118	37.5
	50- above	92	29.2
	**Total**	**315**	**100**
**Gender**	Male	205	65.2
	Female	110	34.8
	**Total**	**315**	**100**
**Education**	Sch. Cert/WAEC	96	30.5
	Bachelors	150	47.7
	Masters	65	20.7
	Pro. Cert/PhD	4	1.1
	**Total**	**315**	**100**
**Experience**	1–5 yrs	76	24.0
	6–10 yrs	166	52.7
	10yrs-above	73	23.3
	**Total**	**315**	**100**

**Table 3:** Standardized total effects (Group number 1 - Default model).

	Entcom	Affective	Busperf	Normative
Affective	.960	.000	.000	.000
Busperf	.174	.000	.000	.000
Normative	.987	.000	.000	.000
MarketS	.157	.000	.905	.000
CustSatis	.199	.000	.803	.060
Profit	.058	-.089	.821	.000
Continuance	.987	.000	.000	.000

**Table 4:** Implied correlations (for all variables).

	Entcom	Affective	Busperf	Normative	MarketS	CustSatis	Profit	Continuance
Entcom	1.000							
Affective	.960	1.000						
Busperf	.174	.167	1.000					
Normative	.987	.947	.172	1.000				
MarketS	.157	.151	.905	.155	1.000			
CustSatis	.199	.191	.813	.198	.735	1.000		
Profit	.058	.049	.806	.057	.729	.651	1.000	
Continuance	.987	.947	.172	.973	.155	.196	.057	1.000

The data shows the correlation estimate between affective commitment and market share (0.151), affective commitment and customers’ satisfaction (0.191), continuance commitment and business performance (0.172), continuance commitment and market share (0.155) and continuance commitment and customers’ satisfaction (0.196).

## 2 Experimental design, materials and methods

This data presentation adopted survey method. The questionnaire is structured and it is designed to gather information from respondents about the influence of commitment [4], [5] towards business performance. 315 copies of the questionnaire were retrieved and coded and input into Statistical Package for Social Science (SPSS) [1], [2], [3]. The analysed was done with Structural Equation Model (SEM). The path analysis as shown in Fig. 3 indicates the estimates of the interconnectedness between the research variables [1].
