# Supplementary material for: Data on modeling of nexus between entrepreneurs׳ commitment and business performance in a developing country
Source: Data Brief. 2018 May 28;19:1068–72. doi: 10.1016/j.dib.2018.05.102 (PMC6139877; doi:10.1016/j.dib.2018.05.102)
Supplement: Supplementary file 1 — Supplementary material [file mmc1.doc]

Ayoade Omisade Ezekiel

Covenant University,

Ota,

Ogun State,

Nigeria

13 April, 2018

The Editor,

Data In Brief,

Dear Sir,

**DECLARATION OF CONFLICT OF INTEREST**

I, Ayoade Omisade Ezekiel and my colleagues write to declare that there is no conflict of interest traceable to our data paper “Data on Modeling of Nexus between Entrepreneurs' Commitment and Business Performance in a Developing Country**”**

Yours faithfully,


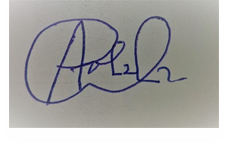


Ayoade Omisade Ezekiel (Corresponding Author)

+234-8037201846
