# Supplementary material for: Data on modeling of nexus between entrepreneurs׳ commitment and business performance in a developing country
Source: Data Brief. 2018 May 28;19:1068–72. doi: 10.1016/j.dib.2018.05.102 (PMC6139877; doi:10.1016/j.dib.2018.05.102)
Supplement: Supplementary file 3 — Supplementary material [file mmc3.docx]

**QUESTIONNAIRE**

**APPENDIX B**

**Bio Data**

**1. Sex:** Female Male

**2. Age:** 20-30yrs 31-40yrs 41-50yrs 51-60yrs 61 years above

**3. Marital Status:** Single Married Divorced Widow Widower

**4. Highest Academic Qualification:** Primary Sch./Secondary Sch. Diploma Studies HND / B.sc / BA M.sc / MBA / MA / MPA PhD Professional Degree

**5. Sector of the economy:** Manufacturing Agriculture Hotel and Restaurants

Educational Service Construction Finance and Business Service

Wholesale and Retail Trade Telecommunication Consulting Services

Others specify…………………………..

**6**. What year has the business been in existence?

1-5yrs 6-10yrs 11-20yrs 21-30yrs 31 years above

**COMMITMENTS**

**Affective Commitment Scale items**

| **S/N** | **Description** | **SA** | **A** | **U** | **D** | **SD** |
| --- | --- | --- | --- | --- | --- | --- |
| **1.** | I would be very happy to spend the rest of my career with this organisation. |  |  |  |  |  |
| **2.** | I enjoy discussing about my organisation with people outside it |  |  |  |  |  |
| **3.** | I really feel as if this organisation’s problems are my own |  |  |  |  |  |

**Continuance Commitment Scale Items**

| **S/N** | **Description** | **SA** | **A** | **U** | **D** | **SD** |
| --- | --- | --- | --- | --- | --- | --- |
| **4.** | I am not afraid of what might happen if I quit my job without having another one lined up. |  |  |  |  |  |
| **5.** | It would be very hard for me to leave my organisation right now, even if I wanted to. |  |  |  |  |  |
| **6.** | Too much in my life would be disrupted if I decided to leave my organisation now. |  |  |  |  |  |

**Normative Commitment Scale Items**

| **S/N** | **Description** | **SA** | **A** | **U** | **D** | **SD** |
| --- | --- | --- | --- | --- | --- | --- |
| **7.** | One of the major reasons I continue to work in this organisation is that I believe loyalty is important and therefore feel a sense of moral obligation to remain. |  |  |  |  |  |
| **8.** | If I got another offer for a better job elsewhere I would not feel it was right to leave my organisation. |  |  |  |  |  |
| **9.** | I was taught to believe in the value of remaining loyal to one organisation. |  |  |  |  |  |

**BUSINESS PERFORMANCE**

**Customers Satisfaction**

| **S/N** | **Description** | **SA** | **A** | **U** | **D** | **SD** |
| --- | --- | --- | --- | --- | --- | --- |
| **1.** | Customers repeat purchase of your products or services is satisfactory. |  |  |  |  |  |
| **2.** | Overall I am very satisfied with the way my customers respond to my product and services. |  |  |  |  |  |
| **3.** | In our business we learn about customers, anticipate their needs, and create business opportunities |  |  |  |  |  |

**Market Share**

| **S/N** | **Description** | **SA** | **A** | **U** | **D** | **SD** |
| --- | --- | --- | --- | --- | --- | --- |
| **4.** | The sales and spread of our products and services are on the increase compared to competitors. |  |  |  |  |  |
| **5.** | Our brand name is becoming more popular and acceptable. |  |  |  |  |  |
| **6.** | The size of our firm is becoming significant in the industry we belong |  |  |  |  |  |

**Profit**

| **S/N** | **Description** | **SA** | **A** | **U** | **D** | **SD** |
| --- | --- | --- | --- | --- | --- | --- |
| **7.** | My organisation is usually satisfied with return on sales. |  |  |  |  |  |
| **8.** | My organisation is usually satisfied with net profit margin. |  |  |  |  |  |
| **9.** | My organisation is usually satisfied with gross profit margin |  |  |  |  |  |
